# Hydrophobia Cured by Oxygen

**Published:** 1879-01

**Authors:** 


Hydrophobia Cured by Oxygen.—This case is reported by Drs. Schmidt and Zebeden from Russia. The first symptoms of rabies appeared seventeen days after the injury. The patient was made to inhale three cubic feet of oxygen, and two hours afterwards he was in a state of perfect calm. Two days afterwards the symptoms of rabies reappeared, and another inhalation of oxygen was administered with the same success. This time the inhalation was continued for forty-five minutes. A slight dyspnœa, which persisted after the disappearance of the graver symptoms, was treated for three weeks by the monobromide of camphor.—*Lyon Medical.*

